# The uric acid/albumin ratio might be a better indicator for predicting repeat revascularization in young patients with acute coronary syndrome: Beyond inflammatory biomarkers

**DOI:** 10.1371/journal.pone.0306178

**Published:** 2024-08-26

**Authors:** Ersan Oflar, Dilay Karabulut, Cennet Yıldız, Hasan Ali Sinoplu, Esra Dönmez, Atilla Koyuncu, Sevgi Özcan, Nihan Turhan Çağlar

**Affiliations:** 1 Department of Cardiology, Bakirkoy Dr Sadi Konuk Training and Research Hospital, Istanbul, Türkiye; 2 Department of Cardiology, Bagcılar Training and Research Hospital, Istanbul, Türkiye; Saud Al-Babtain Cardiac Centre, SAUDI ARABIA

## Abstract

**Background:**

Despite advancements in percutaneous and surgical revascularization techniques, nearly 20% of patients who undergo myocardial revascularization need repeat revascularization. Recently, identified as a prognostication factor for adverse cardiovascular events, the uric acid/albumin ratio (UAR) serves as a new marker for assessing inflammation and oxidative stress. Our objective was to investigate the association between UAR levels and repeat revascularization in young patients with acute coronary syndrome (ACS).

**Methods:**

We enrolled 371 patients with ACS who were under the age of 55 years and who had previously undergone primary percutaneous coronary intervention. Due to their recurrent symptoms, these patients underwent subsequent coronary angiographic examination. The study cohort was splitted into two groups based on whether repeat revascularization was needed.

**Results:**

The study and control groups consisted of 99 and 272 patients, respectively. The mean age of the patients in the study cohort was 41.99±4.99 years. Patients who needed repeat revascularization, in comparison to those who did not, exhibited significantly greater levels of the UAR and uric acid, along with lower levels of neutrophils, stent diameter and high density lipoprotein-cholesterol. Additionally, they had more complex disease, as described by the SYNTAX score. To identify the influential factors associated with repeat revascularization, multivariate logistic regression was performed. SYNTAX score, stent diameter, uric acid levels and the UAR were predictive of the need for repeat revascularization.

**Conclusions:**

UAR was found to be an inexpensive, easily accessible marker for identifying young patients with ACS requiring repeat revascularization.

## Introduction

Improvement in people’s living standards have resulted an increase in the incidence of coronary artery disease (CAD), however the age of the onset of CAD is steadily decreasing. [1]. Myocardial revascularization constitutes the majority of the therapeutic intervention globally [2, 3]. Current percutaneous and surgical revascularization techniques offer outstanding short- and long-term clinical outcomes [2, 3]. In spite of technical and procedural advancements, particularly with the extensive utilization of drug-eluting stents (DES) in modern practice, approximately one-fifth of patients who undergo myocardial revascularization necessitate recurrent revascularization within the initial five years of follow-up [4]. The necessity for repeat revascularization significantly affects the quality of life of patients, exposes them to risks which are linked to hospitalizations, invasive procedures, and substantial economic burdens [4, 5].

With respect to the relations between inflammatory and oxidative biomarkers and atherosclerotic diseases, numerous data have shown that increased uric acid (UA) and high-sensitivity C-reactive protein (hs-CRP) concentrations, which are indicative of systemic inflammation, and reduced anti-inflammatory biomarkers, such as serum albumin (SA), serve as robust predictors of future cardiovascular events [6, 7]. SA may be a factor in the development and progression of atherosclerosis and has a prognostic role in patients with ST-elevation myocardial infarction, dual-chamber permanent pacemakers [8–10]. Many studies have demonstrated a notable correlation between UA concentrations and the occurrence, severeness, and evolution of atherosclerosis. This association has been attributed to the role of UA in the activation of pro-inflammatory pathways and the stimulation of vascular smooth muscle cell proliferation. [11]. The generation and elimination of SA and UA can be influenced by numerous factors, making it difficult to use them as single markers for predicting CAD [12]. The serum uric acid/albumin ratio (UAR), which is based on UA and SA, has also attracted attention in cardiovascular disease research, as studies have demonstrated that combining these parameters with a single index is a more sensitive predictor of inflammation than using UA or SA alone. [12]. UAR has been shown to be predictive of the presence of no-reflow and atrial fibrillation in patients with ST-elevation myocardial infarction (STEMI) [13, 14].

As mentioned above, various studies have shed light on the association between inflammatory parameters and CAD; however, limited data are available concerning the signification of these parameters in young patients with acute coronary syndrome (ACS). Accordingly, we aimed to measure the predictive value of the ratio of these two significant laboratory parameters as the UAR in young patients with ACS. This study provides an overview of whether uric acid/albumin, a simple and easily available inflammatory parameter, predicts repeat myocardial revascularization in young patients with ACS who were treated with DES by focusing on 3 key conditions that lead to repeat revascularization: 1) failure of percutaneous coronary interventions, 2) progression of CAD in native coronary segments previously untreated, and 3) any percutaneous coronary intervention (PCI) or coronary artery bypass surgery (CABG) procedures scheduled to occur after the index PCI. To the best of our knowledge, there are currently no available data on the relationship between UAR and repeat revascularization in patients with young ACS. This information gap limits our understanding of the underlying pathophysiology, as well as the potential implications for risk assessment and prediction in this patient population.

## Methods

### Study population

**Fig 1:**
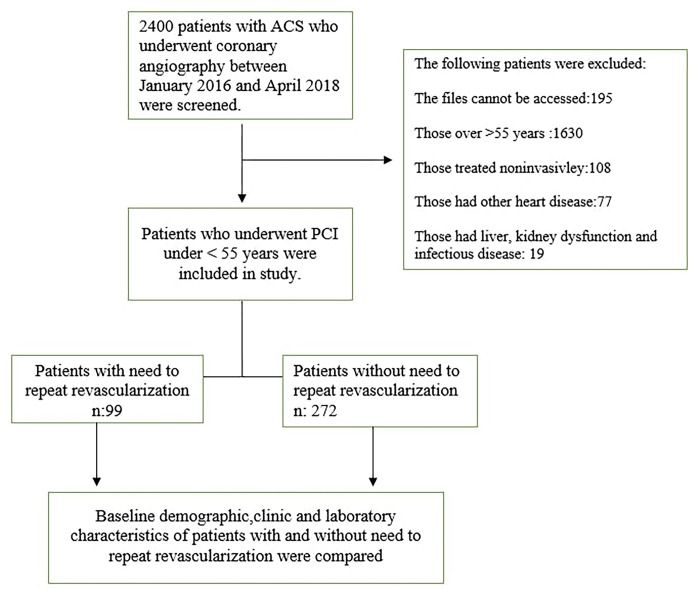
Flowchart of the study.

The medical records of patients with a diagnosis of acute coronary syndrome admitted to our tertiary hospital clinic between 01 January 2016 and 01 April 2018 were reviewed. Data access dates were 2/20/2024 and 3/10/2024. During this period, a total of 2400 patients’ files were obtained. Our exclusion criteria were as follows: (1) medical records that were unavailable for technical reasons; (2) noninvasive treatment; (3) diagnosis of nonischaemic cardiomyopathy, congenital heart disease, or severe valvular disease; (4) liver or kidney dysfunction, malignancy, infection, hypo-hyperthyroidism, or autoimmune diseases; (5) age under 18 or over 55 years; and (6) receiving uric acid-lowering therapy. The files of 195 of the 2400 patients could not be accessed. Of the 2205 patients, 1630 were over 55 years old, 108 were treated noninvasively, 77 had other heart diseases, and 19 had liver or kidney dysfunction and infectious diseases. After applying the exclusion criteria, a total of 371 patients remained and were included in the study. Patients who needed repeat revascularization composed the study group (n = 99), and patients who did not need repeat revascularization composed the control group (n = 272). Data were collected from the hospital records to establish the patients’ initial clinical, demographic and angiographic characteristics. None of the patients were receiving uric acid-lowering therapy. Patients were followed for a mean of 4.32±2.54 years. Patients were followed up through the hospital registry system and by phone. All of the patients were treated in accordance with the current guidelines. Fig 1 shows the flow chart illustrating the selection of the study cohort. Ethical approval was obtained from the Bakirkoy Dr. Sadi Konuk Education and Training Hospital ethical committee (approval number: 2024/58, approval date: 19/02/2024). Written informed consent was obtained from all patients prior to enrollment.

### Angiographic analysis

The Judkins percutaneous transfemoral technique was performed on all patients who underwent PCI by experienced interventional cardiologists. Coronary angiograms of the patients were performed using the Siemens Axiom Artis Zee Cath Lab (Munich, Germany system). Hospital records were used to verify the size of the stents used in the procedures. The severity of coronary artery stenosis was assessed with the SYNTAX anatomical score (SS) using a version downloaded from http://www.syntaxscore.com. All patients were routinely administered 300 mg of aspirin and a 600 mg loading dose of clopidogrel, 180 mg of ticagrelor, or 60 mg of prasugrel before the procedure and received unfractionated heparin during the procedure. The indications for recurrent revascularization included stable angina, ACS, and silent ischemia (detected by stress testing).

The requirement for repeat revascularization frequently arises from progressive atherosclerosis at sites that are not related to the revascularized segment. Target lesion revascularization (TLR) was defined as repeat PCI or bypass graft placement for restenosis occurring within 5 mm of the index PCI site at each site. Stent thrombosis (ST) were assessed independently and were not regarded as TLR events. Repeat revascularization of a coronary artery other than the one treated during the index PCI was accepted as other vessel revascularization. All PCI or CABG procedures planned after completing the index PCI were considered as staged revascularization. Staged procedures accounted for most events within the first 30 days, i.e., other vessel revascularization as unplanned events infrequent during the first month following the index PCI. Presence of ≥50% luminal stenosis within 5 mm proximal or distal to the stent segment or stent region after PCI was accepted as in-stent restenosis.

### Laboratory analysis

When the patient was admitted to the hospital for reperfusion, all laboratory data were collected. Complete blood cell counts were analyzed with an autoanalyzer. Serum UA and SA levels were measured using a Roche Diagnostics Cobas 8000 c502 analyzer (Roche Holding AG, Basel, Switzerland) with normal ranges of 3.4–7.0 mg/L for uric acid and 39–49 g/L for serum albumin. These measurements were taken upon hospital admission. The UAR was obtained by dividing the UA by the SA to get the UAR. Additionally, the monocyte-to-high density lipoprotein-cholesterol (HDL-C) ratio (MHR) and CRP-to-albumin ratio (CAR) were calculated using the complete blood count obtained on admission. Left ventricular ejection fraction (LVEF) was assessed using a modified version of Simpson’s method.

### Statistical analysis

The normality of the data was assessed by analyzing the skewness and kurtosis of the data and by using the Kolmogorov‒Smirnov test. Since all the continuous data were nonparametrically distributed, we expressed them as medians and interquartile ranges. Categorical data are expressed as numbers and percentages. Comparisons between two groups were performed using the Mann‒Whitney U test. Categorical variables were compared by the chi-square test. Receiver operating characteristic (ROC) curve analysis was performed to determine the cutoff values for the uric acid/albumin ratio, MHR and CAR for the prediction of repeat revascularization. Univariate logistic regression analysis was conducted to identify predictors of repeat revascularization. The variables that were found to be significant in the univariate analysis were included in the multivariate logistic regression analysis. A p value less than 0.005 was considered significant.

## Results

The study population consisted of 371 patients who underwent repeat coronary angiography. The mean age of the study population was 41.99±4.99 years, and 43 (11.6%) of them were female.

**Fig 2:**
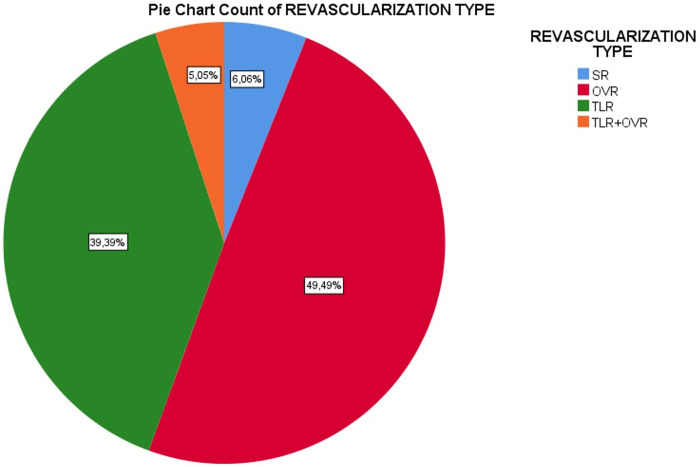
Proportions of all revascularization procedures.

The study and control groups consisted of 99 and 272 patients, respectively. Among the 99 patients in the study group, 39 underwent TLR (39.39%), 6 underwent SR (6.06%), 49 underwent OVR (49.49%), and 5 underwent both TLR and OVR (5.05%). Fig 2 presents the proportions of all revascularization procedures.

**Fig 3:**
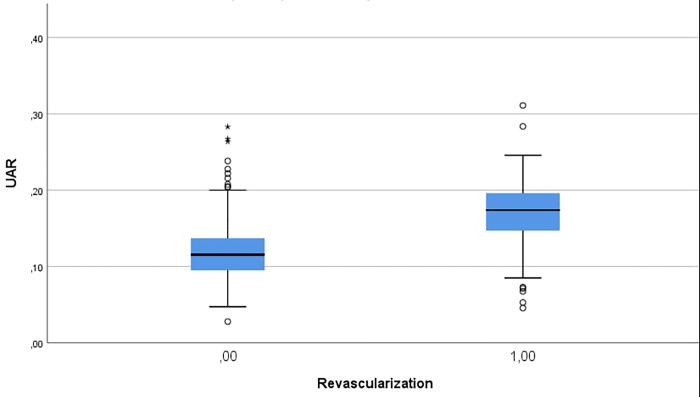
UAR of patients who did not need repeat revascularization and those who did.

Patients who required repeat revascularization, compared with those who did not, had a significantly greater UAR (Fig 3) and UA (p<0.001, for both), lower neutrophil, stent diameter and HDL-C levels (p = 0.042, p = 0.001, p = 0.031, respectively) and more complex disease, as described by the SS (13.25 (8–18.5) and 19 (11–23.5), respectively; p<0.001). The total mortality rate of the patients was 6.3%, and mortality was greater in those with a high UAR (0.13±0.11 and 0.19±0.06, respectively, p<0.001). In the group that underwent repeated revascularization, the percentage of patients with non-ST-elevation myocardial infarction (NSTEMI) was 30.3%, the percentage of patients with STEMI was 61.6%, and the percentage of patients with unstable angina (UA) was 8.1%. There was no significant difference in the MHR or CAR between the two groups (p = 0.471, p = 0.060, p>0.001, respectively). At the end of the follow-up period, 223 (89.9%) of the patients in the control group and 82 (87.2%) of the patients in the study group were using acetylsalicylic acid. There were no significant differences in P2Y12 use between the two groups at one year or at the end of follow-up. The baseline clinical characteristics of the two groups, such as age, laboratory parameters, cardiovascular risk factors, and current medications on admission, are listed in Table 1.

**Table 1:** Demographic, clinical and laboratory characteristics of the patients.

	Control group (n = 272)	Study group (n = 99)	p
Age (years)	43 (39–45)	44 (39–45)	0.858
Gender (n, %)			0.848
Male	241 (88.6)	87 (87.9)	
Female	31 (11.4)	12 (12.1)	
Diabetes, n (%)	73 (26.9)	38 (38.4)	0.228
Hypertension, n (%)	97 (35.8)	15 (26.8)	0.647
Family history of CAD, n (%)	124 (48.1)	56 (58.3)	0.086
Stroke/ TIA, n (%)	5 (1.9)	4 (4.1)	0.252
PAD, n (%)	7 (1.9)	1 (1)	0.687
P2Y12 use, n (%) (at one year)			0.419
Clopidogrel	40 (15.2)	19 (19.8)	
Ticagrelor	147 (55.7)	51 (53.1)	
Prasurgel	72 (27.3)	26 (27.1)	
ASA use, n (%) (end of follow-up)	223 (89.9)	82 (87.2)	0.482
P2Y12 use, n (%) (end of follow up)			0.086
Clopidogrel	50 (20.2)	25 (26.6)	
Ticagrelor	52 (21.1)	21 (22.3)	
Prasurgel	9 (3.6)	8 (8.5)	
Statin use, n (%)			0.543
No use	28 (10.3)	11 (11.1)	
Low dose	33 (12.1)	8 (8.1)	
High dose	211 (77.6)	80 (80.8)	
LVEF(%)	53 (45–60)	50 (45–58)	0.879
Glucose (mg/dl)	112 (98–140)	117 (97.25–161.75)	0.684
HgA1c	5.7 (5.4–6.3)	5.8 (5.4–6.92)	0.487
Hemoglobin (mg/dl)	14.6 (13.5–15.5)	14.3 (13.2–15.59)	0.418
RDW (%)	13.3 (12.4–13.9)	12.95 (12.3–13.72)	0.193
Leukocyte count/1000,	11.43 (9.3–14.68)	10.90 (9.1–13.45)	0.110
Neutrophil count /1000,	7.68 (5.71–10.60)	7.0 (5.51–8.9)	0.042
Lymphocyte count /1000,	2.45 (1.9–3.29)	2.76 (2.06–3.41)	0.147
Eosinophil count /1000,	0.14 (0.07–0.24)	0.14 (0.07–0.24)	0.912
Monocyte count /1000,	0.67 (0.52–0.93)	0.67 (0.50–0.90)	0.636
Platelet count 1000/ml	245 (210–296.5)	235 (176–262)	0.232
Creatinine (mg/dl)	0.81 (0.71–0.93)	0.80 (0.70–0.93)	0.551
Total cholesterol (mg/dl)	197 (165–228.5)	195 (160–236)	0.961
Triglycerides (mg/dl)	156 (104–236)	146 (107–226)	0.891
HDL-C (mg/dl)	37 (32–44)	34 (30–43)	0.031
LDL-C (mg/dl)	122 (99–156.25)	135 (96.25–154)	0.391
CRP (mg/dl) (IQR)	6 (3–12)	7 (4–14.0)	0.124
Uric acid (mg/dl)	4.9 (4.1–5.8)	7.3 (6–8)	<0.001
Albumin (g/dl)	42.75 (40.02–45.5)	41 (39–43.4)	0.001
Monocyte/HDL-C ratio	0.018 (0.013–0.025)	0.019 (0.014–0.026)	0.471
CRP/albumin ratio,	0.13 (0.06–0.27)	0.16 (0.09–0.38)	0.060
UAR	0.11 (0.09–0.13)	0.17 (0.14–0.19)	<0.001
Stent length, (mm)	23 (18–28)	23 (16–25)	0.979
Stent diameter, (mm)	3.01±0.37	2.78±0.38	0.001
SYNTAX score	13.25 (8–18.5)	19 (11–23.5)	<0.001
Type of ACS (n;%)			0.691
UA	19 (7)	8 (8.1)	
NSTEMI	95 (34.9)	30 (30.3)	
STEMI	158 (58.1)	61 (61.6)	

CRP: C-reactive protein;HDL-C: high-density lipoprotein cholesterol; IQR: interquartile range (25th-75th percentiles); LDL-C: low-density lipoprotein cholesterol; LVEF: left ventricular ejection fraction; RDW: red distrubution width;ACS:Acute coronary syndrome; STEMI: ST-segment elevation myocardial infarction; NSTEMI: Non ST segment elevation myocardial infarction;UA:Unstabil angina, UAR: Uric acid albumin ratio, TIA: Transient ischemic attack, PAD: peripheral arterial disease.

**Fig 4:**
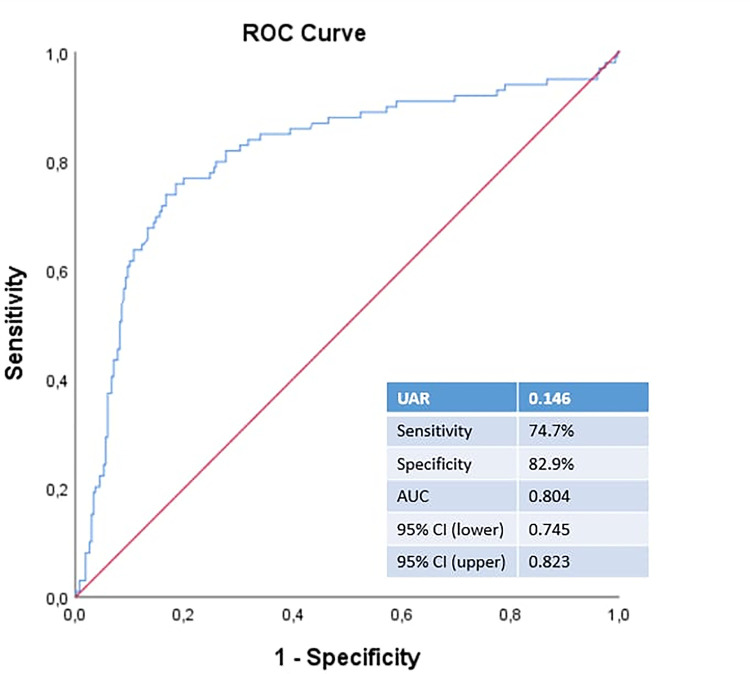
ROC curve analysis of UAR for prediction of repeat revascularization.

Patients were also divided into two groups: those with and without ISR. SR was observed in 44 (11.9%) out of 371 patients in our cohort. The UAR was greater in the ISR group than in the non-ISR group (0.17±0.05 and 0.13±0.11, respectively, p<0.001) (Table 2). ROC curve analysis revealed that the cutoff value for the UAR that indicated the need for repeat revascularization was 0.146, with 74.7% sensitivity and 82.9% specificity (Fig 4).

**Table 2:** Laboratory and angiographic characteristics of patients with and without restenosis.

	Patient without restenosis (n = 327)	Patients with restenosis (n = 44)	p
UAR	0.13±0.11	0.17±0.05	<0.001
Monocyte/HDL-C ratio	0.04±0.29	0.02±0.01	0.569
CRP/albumin ratio	0.40±1.21	0.35±0.69	0.855
Stent length (mm)	22.38±6.35	21.02±5.98	0.197
Stent diameter (mm)	2.98±0.36	2.90±0.44	0.121

UAR: Uric acid albumin ratio, CRP: C-reactive protein, HDL-C: High density lipoprotein-cholesterol.

To determine the role of the UAR in predicting repeat revascularization, we performed univariate and multivariate logistic regression modeling. According to the univariate analysis, the SS, stent diameter, uric acid concentration, serum ALB concentration and UAR were found to be independent predictors of repeat revascularization. Since the UAR includes both uric acid and albumin, two separate logistic regression analyses were performed. In Model A and Model B, the UAR and uric acid and albumin levels were tested. The results of multivariate logistic regression showed that in addition to the SS and stent diameter, the uric acid level and UAR were predictive of the need for repeat revascularization. The UAR had a greater OR than did the uric acid level, indicating stronger discriminative power. (Table 3).

**Table 3:** Univariate and multivariate logistic regression analyses for prediction of repeat revascularization.

	Univariate analysis			Multivariate analysis					
				Model A			Model B		
	p	OR	95% CI	p	OR	95% CI	p	OR	95% CI
Neutrophil	0.052	0.996	0.991–1.000						
HDL-C	0.110	0.980	0.955–1.005						
SYNTAX score	<0.001	1.059	1.029–1.090	0.022	1.039	1.006–1.074	0.012	1.028	1.007–1.066
Stent diameter	0.006	0.366	0.178–0.751	0.012	0.387	0.185–0.811	0.009	0.353	0.161–0.771
Uric acid	<0.001	2.109	1.760–2.626				0.001	1.942	1.601–2.356
Albumin	0.017	0.948	0.908–0.990				0.074	0.953	0.904–1.005
UAR	<0.001	4.961	0.129–9.794	0.001	3.038	1.867–5.056			

CI: confidence interval; HDL-C: high-density lipoprotein cholesterol; OR: odds ratio; UAR: uric acid/albümin ratio

## Discussion

This study revealed that the UAR was not only associated with but also an independent predictor of repeat revascularization in young patients with ACS undergoing PCI. The UAR was better than both the MHR and the CAR in predicting repeat revascularization in young patients with ACS. In addition, the UAR was found to be high in young patients with ACS who had stent restenosis and a high SYNTAX score.

In the initial era of PCI with bare-metal stents, restenosis was the predominant cause for repeat procedures within the first year post-PCI, whereas the treatment of nontarget lesions became more prevalent in subsequent follow-up [15]. Our analysis revealed that repeat revascularization was performed in ≈12% of individuals by 4.32±2.54 years of follow-up. Similarly, in a study conducted by Stolker et al., the repeat revascularization rate was 11.9% [16]. The etiology of repeat revascularization is complex and multifactorial, including biological or patient-related, clinical and technical factors. A growing body of evidence indicates that inflammation significantly contributes to promoting neointimal growth and subsequent luminal narrowing [17]. Although UA has been identified as an antioxidant in experimental investigations, it is also acknowledged that UA can induce inflammation in vascular endothelial and smooth muscle cells in addition to causing intracellular oxidative stress, consequently resulting in endothelial dysfunction [18]. Several studies have shown that elevated serum UA levels were associated with coronary atherosclerosis, more severe CAD, increased cardiovascular and all-cause mortality and predicted poor coronary collateralization in patients with stable angina pectoris [19, 20]. The SA concentration decreases during inflammatory processes, and SA provides antioxidant protection [21].To date, numerous studies have demonstrated that low SA levels are correlated with increased cardiovascular morbidity and mortality across a broad spectrum of cardiovascular diseases [21, 22]. In our study, basal UAR and UA levels were higher in young ACS patients who underwent repeat revascularization. In contrast to our findings Sultana et al. found that there was no correlation between UAR and severity of CAD as assessed by SS. In their study, the mean age of the study population was 61.43 years, and the long-term value of UAR was not evaluated [23].

Recent studies have emphasized numerous new combined markers, such as the MHR and CAR, as better independent indicators of inflammatory status and as predictors of outcomes in patients with STEMI [24, 25]. The associations of the MHR with the progression and presence of CVD have been revealed by many studies [24, 26]. Monocytes can secrete both proinflammatory and prooxidant cytokines. HDL-C functions in transporting cholesterol from peripheral tissues to the liver and has been shown to participate in anti-inflammatory, antioxidant, and antithrombotic processes [27]. Similarly, there is considerable evidence that higher baseline CRP levels are related to an increased risk of major adverse cardiovascular outcomes [28]. In contrast with previous studies, in our study, although the MHR and CAR were high in the repeat revascularization group, they did not reach statistical significance; only the UAR was found to be a statistically significant predictor of recurrent myocardial revascularization in young ACS patients. In a study performed by Ulucay et al. high sensitive CRP levels did not predict the presence and severity of CAD in patients with stable angina [29]. Although albumin was used in the calculation of both CAR and UAR, only UAR had predictive power for the presence of recurrent revascularization in young ACS patients suggesting that UA was an effective component in these formulas. Therefore, among these inflammatory-based markers, UAR appears to be the most valuable in this study. The response to inflammatory events may not be uniform among the various acute phase reactants. In diseases where inflammation plays a critical role, combining UA and SA into a single index that provides stability between between fluctuating UA and SA levels. Consistent with these findings our multivariant analysis revealed that the ratio of UA to SA was a better predictor of inflammatory status than was UA alone.

Our study also showed that patients who underwent repeat revascularization had significantly lower HDL-C levels than those who did not. Elevated levels of HDL are recognized for their protective role against atherosclerosis and are associated with a better prognosis for events related to vascular disease [30].

In contrast to its decline in other age groups, cardiovascular disease remains a prominent cause of adverse outcomes in young people. Compared to older populations, this population has a distinct risk profile with fewer traditional cardiovascular risk factors. [31]. Consequently, in recent years, there has been a notable occurrence of CAD in individuals under the age of 50, and that is classified as early-onset CAD. Early- and late-onset CAD have different etiologies and risk factors [31]. This gap in the literature was the impetus for our investigation of the potential relationship between preprocedural UAR and the need for repeat revascularization in young ACS patients. Consequently, individuals with higher UAR levels may have greater susceptibility to inflammation, oxidative stress and severe CAD than those with lower UAR levels [12, 32, 33]. As some researchers’ suggestions, the role of more aggressive monitoring of the progression of atherosclerosis and potentially more intensive anti-inflammatory treatments to decrease disease progression should be investigated in future studies, especially in young patients.

## Conclusion

This study suggested that a high UAR on admission was independently associated with repeat revascularization and stent restenosis in young patients with ACS. SS is also related to the severity and complexity of coronary atherosclerosis. It may be part of the cardiovascular assessment to identify young individuals with ACS who are at high risk of advanced CAD. These individuals may necessitate a more aggressive treatment approach and closer clinical monitoring. For this reason, UAR assessment can be considered for early risk stratification of young patients with ACS.

### Limitations

Our investigation has a few limitations. First, the retrospective methodology of the present study limits its ability to definitively establish a direct causative link between UAR and the need for repeat revascularization. Second, the laboratory parameters were only measured at the time of admission, with no serial follow-up. Third, since some of the patients who underwent repeated revascularization underwent intervention at an external center, the effect of patient compliance and continuity of treatment on these results is not clear.
